# Supplementary material for: Association between Toxoplasma gondii seropositivity and serointensity and brain volume in adults: A cross-sectional study
Source: PLoS One. 2021 Feb 5;16(2):e0245994. doi: 10.1371/journal.pone.0245994 (PMC7864421; doi:10.1371/journal.pone.0245994)
Supplement: S1 File — (DOCX) [file pone.0245994.s001.docx]

| S1 Table.  Adjusted models^a^ of brain region volume (in mm^3^) on the interaction of *Toxoplasma gondii* and age:  Unstandardized coefficients from linear regression | | | | | | | | | | |  |  |
| --- | --- | --- | --- | --- | --- | --- | --- | --- | --- | --- | --- | --- |
|  | Frontal | | | | | |  | | |  | |  |
|  | Pole | Superior  gyrus | Medial  cortex | Orbital  cortex | Operculum  cortex | Hippocampus | | Thalamus | Multivariate  *p*^b^ | | |  |
| Seropositive |  |  |  |  |  |  | |  | .016 | | |  |
| Left |  |  |  |  |  |  | |  |  | | |  |
| Toxo | -4696.85 | -2884.57 | -323.17 | -87.32 | 208.73 | -488.98 | | 180.20 |  | | |  |
| Age | -90.10*** | -14.07 | -7.78* | -28.78*** | -5.96** | -12.57** | | -22.85*** |  | | |  |
| Toxo x Age | 68.93 | 44.66 | 4.63 | 1.24 | -3.52 | 6.19 | | -1.67 |  | | |  |
| Right |  |  |  |  |  |  | |  |  | | |  |
| Toxo | -3739.61 | -1346.95 | -559.03 | 110.30 | -131.78 | -237.32 | | 593.24 |  | | |  |
| Age | -62.79* | -3.41 | -8.82** | -28.81*** | -4.16 | -4.93 | | -18.58*** |  | | |  |
| Toxo x Age | 54.60 | 16.62 | 8.58 | -.76 | 1.57 | 2.69 | | -8.68 |  | | |  |
| ln(p22) |  |  |  |  |  |  | |  | .002 | | |  |
| Left |  |  |  |  |  |  | |  |  | | |  |
| Toxo | -724.37 | -768.89 | -16.23 | -282.19 | 50.49 | -146.94 | | -54.47 |  | | |  |
| Age | -112.20* | -45.78 | -7.67 | -43.29** | -4.09 | -18.49* | | -27.13* |  | | |  |
| Toxo x Age | 10.46 | 12.34 | .25 | 4.30 | -.78 | 1.95 | | 1.43 |  | | |  |
| Right |  |  |  |  |  |  | |  |  | | |  |
| Toxo | -1197.12 | -632.21 | -131.21 | 28.04 | -72.00 | -214.44 | | 62.03 |  | | |  |
| Age | -113.46* | -32.09 | -14.20* | -28.03* | -7.56 | -15.87 | | -17.84 |  | | |  |
| Toxo x Age | 18.03 | 9.00 | 2.19 | -.11 | 1.06 | 3.36 | | -.61 |  | | |  |
| ln(sag1) |  |  |  |  |  |  | |  | .049 | | |  |
| Left |  |  |  |  |  |  | |  |  | | |  |
| Toxo | -902.32 | -1150.40 | 24.35 | -551.90 | 114.97 | 301.72 | | 230.28 |  | | |  |
| Age | -127.97 | -86.40 | -4.61 | -65.29* | 1.24 | 11.31 | | -9.35 |  | | |  |
| Toxo x Age | 12.06 | 18.81 | -.50 | 8.43 | -1.83 | -5.20 | | -3.15 |  | | |  |
| Right |  |  |  |  |  |  | |  |  | | |  |
| Toxo | -952.00 | -288.66 | -111.70 | -324.39 | -19.50 | 182.20 | | 477.01 |  | | |  |
| Age | -114.10 | -16.20 | -15.79 | -51.92* | -5.63 | 10.24 | | 11.30 |  | | |  |
| Toxo x Age | 14.41 | 3.66 | 2.02 | 5.28 | .40 | -3.36 | | -7.27 |  | | |  |
| Mean of ln(p22) and ln(sag1) |  |  |  |  |  |  | |  | .005 | | |  |
| Left |  |  |  |  |  |  | |  |  | | |  |
| Toxo | -1283.77 | -1444.19 | -3.39 | -605.05 | 117.12 | -2.93 | | 83.44 |  | | |  |
| Age | -75.55*** | -2.78 | -6.84* | -28.19*** | -6.86** | -11.69** | | -22.75*** |  | | |  |
| Toxo x Age | 18.10 | 23.33 | -.02 | 9.24 | -1.85 | -.52 | | -.58 |  | | |  |
| Right |  |  |  |  |  |  | |  |  | | |  |
| Toxo | -1822.50 | -860.33 | -198.56 | -135.90 | -90.95 | -127.76 | | 336.47 |  | | |  |
| Age | -50.29* | -.28 | -6.65* | -28.55*** | -3.80 | -4.54 | | -20.47*** |  | | |  |
| Toxo x Age | 27.66 | 12.23 | 3.38 | 2.51 | 1.43 | 1.67 | | -4.87 |  | | |  |
| Note: ^a^ Each set of three coefficients represents results from a single model that contains a single interaction. The coefficients reported are the main effect for the *T. gondii* measure, the main effect for the predictor, and the interaction between the *T. gondii* measure and predictor. For example, the first entry in the table is for the interaction between *T. gondii* seropositivity and age with volume of the frontal pole as the outcome. Each model adjusts for included sex, race, education, income, self-rated health, body-mass index, smoking status, and drinking frequency, although these coefficients are not reported. ^b^ N = 385. Source: *UK Biobank*. * p < .05. ** p < .01. *** p < .001. | | | | | | | | | | |  |  |

| S2 Table.  Adjusted models ^a^ of brain region volume (in mm^3^) on the interaction of *Toxoplasma gondii* and sex:  Unstandardized coefficients from linear regression | | | | | | | | | |
| --- | --- | --- | --- | --- | --- | --- | --- | --- | --- |
|  | Frontal | | | | |  |  |  |  |
|  | Pole | Superior  gyrus | Medial  cortex | Orbital  cortex | Operculum  cortex | Hippocampus | Thalamus | Multivariate  *p* |  |
| Seropositive |  |  |  |  |  |  |  | .546 |  |
| Left |  |  |  |  |  |  |  |  |  |
| Toxo | -391.75 | -315.62 | 45.71 | -48.17 | -33.61 | -99.55 | 37.48 |  |  |
| Female | -2259.59*** | -1147.96*** | -118.47** | -637.44*** | -138.30*** | -231.38*** | -722.43*** |  |  |
| Toxo x Female | -21.08 | 369.14 | -139.91 | 65.73 | 40.56 | -7.09 | 67.02 |  |  |
| Right |  |  |  |  |  |  |  |  |  |
| Toxo | -292.84 | -541.80* | 16.58 | -86.41 | -63.68 | -127.94 | 15.08 |  |  |
| Female | -2520.85*** | -1047.98*** | -74.58 | -680.50*** | -142.90*** | -266.50*** | -767.70*** |  |  |
| Toxo x Female | -81.09 | 399.59 | -72.21 | 260.54 | 51.91 | 101.42 | 65.07 |  |  |
| ln(p22) |  |  |  |  |  |  |  | .441 |  |
| Left |  |  |  |  |  |  |  |  |  |
| Toxo | -43.30 | -57.76 | 17.38 | .34 | -9.66 | 12.47 | 34.86 |  |  |
| Female | -2057.05** | -1327.04** | -51.82 | -514.40* | -198.66** | -10.81 | -696.51*** |  |  |
| Toxo x Female | -68.25 | 79.83 | -31.11 | -32.44 | 21.10 | -68.97 | -2.80 |  |  |
| Right |  |  |  |  |  |  |  |  |  |
| Toxo | 21.72 | -139.63 | 12.98 | 13.01 | -1.60 | 28.99 | 23.56 |  |  |
| Female | -1914.47* | -1300.12** | -37.96 | -665.52*** | -100.00 | -34.77 | -756.26*** |  |  |
| Toxo x Female | -194.56 | 102.78 | -16.99 | 14.68 | -9.76 | -64.57 | 1.35 |  |  |
| ln(sag1) |  |  |  |  |  |  |  | .119 |  |
| Left |  |  |  |  |  |  |  |  |  |
| Toxo | -230.72 | -53.45 | 6.13 | -18.53 | -3.70 | -4.95 | -5.17 |  |  |
| Female | -2972.90** | -1742.11* | -35.26 | -542.44 | -169.03 | -83.16 | -1057.98*** |  |  |
| Toxo x Female | 162.74 | 153.45 | -26.78 | -17.18 | 9.04 | -34.83 | 80.01 |  |  |
| Right |  |  |  |  |  |  |  |  |  |
| Toxo | -1.69 | -96.87 | 19.13 | -43.54 | 20.36 | -37.57 | -12.19 |  |  |
| Female | -2081.77 | -1284.75 | -52.39 | -1048.45*** | -.55 | -332.71 | -1077.59*** |  |  |
| Toxo x Female | -107.34 | 73.88 | -9.94 | 98.93 | -30.48 | 20.80 | 74.12 |  |  |
| Mean of ln(p22) and ln(sag1) |  |  |  |  |  |  |  | .605 |  |
| Left |  |  |  |  |  |  |  |  |  |
| Toxo | -187.79 | -76.24 | 15.98 | -12.52 | -8.98 | 4.73 | 19.86 |  |  |
| Female | -2262.42*** | -1053.63*** | -156.65*** | -621.53*** | -127.68*** | -241.65*** | -703.64*** |  |  |
| Toxo x Female | 53.37 | 154.38 | -39.85 | -35.99 | 21.85 | -76.46 | 52.45 |  |  |
| Right |  |  |  |  |  |  |  |  |  |
| Toxo | 12.41 | -161.10 | 21.74 | -21.06 | 12.77 | -6.31 | 7.43 |  |  |
| Female | -2567.22*** | -948.35*** | -96.59* | -610.61*** | -135.10*** | -246.67*** | -749.13*** |  |  |
| Toxo x Female | -224.59 | 115.21 | -19.71 | 78.12 | -28.67 | -33.46 | 51.03 |  |  |
| Note: ^a^ Each set of three coefficients represents results from a single model that contains a single interaction. The coefficients reported are the main effect for the *T. gondii* measure, the main effect for the predictor, and the interaction between the *T. gondii* measure and predictor. For example, the first entry in the table is for the interaction between *T. gondii* seropositivity and sex with volume of the frontal pole as the outcome. Each model adjusts for included age, race, education, income, self-rated health, body-mass index, smoking status, and drinking frequency, although these coefficients are not reported. N = 385. Source: *UK Biobank*. * p < .05. ** p < .01. *** p < .001. | | | | | | | | | |

| S3 Table.  Adjusted models ^a^ of brain region volume (in mm^3^) on the interaction of *Toxoplasma gondii* and education:  Unstandardized coefficients from linear regression | | | | | | | | | | |  |
| --- | --- | --- | --- | --- | --- | --- | --- | --- | --- | --- | --- |
|  | Frontal | | | | |  | |  | |  | |
|  | Pole | Superior  gyrus | Medial  cortex | Orbital  cortex | Operculum  cortex | Hippocampus | Thalamus | | Multivariate  *p* | |  |
| Seropositive |  |  |  |  |  |  |  | | .020 | |  |
| Left |  |  |  |  |  |  |  | |  | |  |
| Toxo | -793.42 | 396.77 | -42.43 | -40.55 | -23.56 | -181.12* | -70.14 | |  | |  |
| College degree | 388.68 | 550.51** | -12.47 | 169.28 | 39.79 | 35.57 | 104.74 | |  | |  |
| Toxo x College degree | 759.82 | -975.85** | 15.11 | 58.81 | 25.85 | 151.16 | 285.01* | |  | |  |
| Right |  |  |  |  |  |  |  | |  | |  |
| Toxo | -654.01 | -251.89 | -39.67 | -48.56 | -18.58 | -98.34 | -62.25 | |  | |  |
| College degree | 308.94 | -7.65 | -21.84 | 75.57 | -4.76 | 1.22 | 82.26 | |  | |  |
| Toxo x College degree | 613.58 | -117.66 | 28.81 | 218.15 | -29.79 | 55.92 | 223.76 | |  | |  |
| ln(p22) |  |  |  |  |  |  |  | | .189 | |  |
| Left |  |  |  |  |  |  |  | |  | |  |
| Toxo | -271.79 | 78.54 | 7.51 | -41.94 | -9.20 | -43.71 | -15.06 | |  | |  |
| College degree | -432.22 | 777.90 | 37.92 | 55.40 | -17.42 | -9.51 | -83.46 | |  | |  |
| Toxo x College degree | 310.55 | -148.06 | -13.37 | 38.63 | 19.31 | 26.73 | 79.36 | |  | |  |
| Right |  |  |  |  |  |  |  | |  | |  |
| Toxo | -173.55 | -117.85 | 15.88 | -10.69 | -1.11 | -40.88 | -16.62 | |  | |  |
| College degree | 32.02 | -241.61 | 57.64 | -42.46 | 21.71 | -160.66 | -82.15 | |  | |  |
| Toxo x College degree | 135.86 | 61.95 | -20.92 | 52.90 | -10.09 | 53.41 | 67.31 | |  | |  |
| ln(sag1) |  |  |  |  |  |  |  | | .132 | |  |
| Left |  |  |  |  |  |  |  | |  | |  |
| Toxo | -277.16 | 198.75 | 1.91 | -49.59 | -25.82 | -24.48 | -4.23 | |  | |  |
| College degree | -282.36 | 1516.14* | 53.42 | 24.36 | -136.90 | 60.60 | -77.54 | |  | |  |
| Toxo x College degree | 197.31 | -277.97 | -13.87 | 35.54 | 41.59 | 3.97 | 59.74 | |  | |  |
| Right |  |  |  |  |  |  |  | |  | |  |
| Toxo | -263.72 | -30.06 | 14.87 | -44.82 | 1.38 | -37.61 | -2.09 | |  | |  |
| College degree | -972.50 | 174.55 | -6.51 | -209.15 | -39.56 | -55.74 | -34.28 | |  | |  |
| Toxo x College degree | 328.65 | -48.21 | -.93 | 77.69 | 6.43 | 15.97 | 40.87 | |  | |  |
| Mean of  ln(p22) and ln(sag1) |  |  |  |  |  |  |  | | .086 | |  |
| Left |  |  |  |  |  |  |  | |  | |  |
| Toxo | -387.12 | 183.36 | 7.19 | -63.89 | -23.00 | -50.19 | -14.47 | |  | |  |
| College degree | 609.69* | 273.50 | -7.98 | 184.40* | 48.68 | 78.78 | 189.18** | |  | |  |
| Toxo x College degree | 364.52 | -286.98 | -19.33 | 52.23 | 40.81 | 23.97 | 99.06 | |  | |  |
| Right |  |  |  |  |  |  |  | |  | |  |
| Toxo | -299.76 | -113.63 | 21.82 | -35.58 | -.09 | -55.74 | -14.50 | |  | |  |
| College degree | 490.71 | -39.03 | -12.18 | 138.75 | -12.23 | 18.33 | 148.51* | |  | |  |
| Toxo x College degree | 312.44 | 20.48 | -16.64 | 88.80 | -3.40 | 51.25 | 77.96 | |  | |  |
| Note: ^a^ Each set of three coefficients represents results from a single model that contains a single interaction. The coefficients reported are the main effect for the *T. gondii* measure, the main effect for the predictor, and the interaction between the *T. gondii* measure and predictor. For example, the first entry in the table is for the interaction between *T. gondii* seropositivity and education with volume of the frontal pole as the outcome. Each model adjusts for included age, sex, race, income, self-rated health, body-mass index, smoking status, and drinking frequency, although these coefficients are not reported. N = 385. Source: *UK Biobank*. * p < .05. ** p < .01. *** p < .001. | | | | | | | | | | |  |

| S4 Table.  Adjusted models ^a^ of brain region volume (in mm^3^) on the interaction of *Toxoplasma gondii* and income (in 10,000 ₤.):  Unstandardized coefficients from linear regression | | | | | | | | | | | |  |  |
| --- | --- | --- | --- | --- | --- | --- | --- | --- | --- | --- | --- | --- | --- |
|  | Frontal | | | | | |  | |  | |  | | |
|  | Pole | Superior  gyrus | Medial  cortex | Orbital  cortex | Operculum  cortex | Hippocampus | | Thalamus | | Multivariate  *p* | | |  |
| Seropositive |  |  |  |  |  |  | |  | | .167 | | |  |
| Left |  |  |  |  |  |  | |  | |  | | |  |
| Toxo | 21.28 | 329.40 | -.32 | 179.05 | 33.02 | -184.05 | | 63.69 | |  | | |  |
| Income | 61.42 | 26.26 | 9.34 | 15.41 | -2.68 | -4.83 | | 1.10 | |  | | |  |
| Toxo x Income | -95.36 | -97.10 | -7.71 | -42.49 | -9.72 | 18.04 | | 2.76 | |  | | |  |
| Right |  |  |  |  |  |  | |  | |  | | |  |
| Toxo | 547.93 | 65.75 | 75.60 | 161.35 | 6.50 | -62.30 | | 39.31 | |  | | |  |
| Income | 163.65** | -.92 | 17.83* | 18.12 | 8.22 | 15.28 | | 2.67 | |  | | |  |
| Toxo x Income | -199.03 | -84.78 | -22.54 | -22.00 | -9.05 | -1.65 | | 2.95 | |  | | |  |
| ln(p22) |  |  |  |  |  |  | |  | | .242 | | |  |
| Left |  |  |  |  |  |  | |  | |  | | |  |
| Toxo | -51.84 | -6.00 | 5.11 | .16 | 12.45 | -42.80 | | 47.28 | |  | | |  |
| Income | 48.58 | -2.87 | 10.63 | 14.30 | 1.07 | -10.57 | | 12.68 | |  | | |  |
| Toxo x Income | -6.39 | -1.15 | -1.19 | -3.84 | -2.04 | 3.17 | | -2.90 | |  | | |  |
| Right |  |  |  |  |  |  | |  | |  | | |  |
| Toxo | 58.48 | -63.99 | 39.00 | 28.68 | -21.09 | -32.68 | | 29.24 | |  | | |  |
| Income | 200.84 | -20.14 | 35.67* | 16.42 | -4.90 | -3.05 | | 7.61 | |  | | |  |
| Toxo x Income | -30.85 | -3.34 | -7.41 | -1.48 | 2.86 | 5.02 | | -1.01 | |  | | |  |
| ln(sag1) |  |  |  |  |  |  | |  | | .346 | | |  |
| Left |  |  |  |  |  |  | |  | |  | | |  |
| Toxo | 10.66 | 97.85 | 8.90 | 6.27 | 4.76 | -67.55 | | 5.79 | |  | | |  |
| Income | 192.43 | 70.93 | 22.70 | 35.06 | -1.87 | -45.84 | | -26.20 | |  | | |  |
| Toxo x Income | -36.90 | -17.41 | -3.61 | -7.56 | -.92 | 10.39 | | 6.40 | |  | | |  |
| Right |  |  |  |  |  |  | |  | |  | | |  |
| Toxo | 55.58 | 54.74 | 32.73 | 6.53 | -4.89 | -62.58 | | -17.54 | |  | | |  |
| Income | 206.95 | 86.65 | 28.88 | 13.01 | -5.60 | -21.42 | | -38.37 | |  | | |  |
| Toxo x Income | -24.99 | -26.33 | -4.20 | -.41 | 2.36 | 8.01 | | 9.46 | |  | | |  |
| Mean of ln(p22) and ln(sag1) |  |  |  |  |  |  | |  | | .220 | | |  |
| Left |  |  |  |  |  |  | |  | |  | | |  |
| Toxo | -12.32 | 67.26 | 10.42 | 6.85 | 11.27 | -75.55 | | 31.28 | |  | | |  |
| Income | 27.71 | -6.47 | 6.62 | 1.36 | -5.94 | .31 | | 2.44 | |  | | |  |
| Toxo x Income | -32.93 | -14.02 | -3.41 | -8.52 | -1.97 | 8.99 | | 3.57 | |  | | |  |
| Right |  |  |  |  |  |  | |  | |  | | |  |
| Toxo | 81.21 | 5.16 | 47.49 | 22.62 | -17.35 | -64.77 | | 3.36 | |  | | |  |
| Income | 96.01 | -30.70 | 10.32 | 11.22 | 4.91 | 14.08 | | 3.85 | |  | | |  |
| Toxo x Income | -41.56 | -23.66 | -8.04 | -.64 | 3.38 | 9.14 | | 6.86 | |  | | |  |
| Note: ^a^ Each set of three coefficients represents results from a single model that contains a single interaction. The coefficients reported are the main effect for the *T. gondii* measure, the main effect for the predictor, and the interaction between the *T. gondii* measure and predictor. For example, the first entry in the table is for the interaction between *T. gondii* seropositivity and income with volume of the frontal pole as the outcome. Each model adjusts for included age, sex, race, education, self-rated health, body-mass index, smoking status, and drinking frequency, although these coefficients are not reported. N = 385. Source: *UK Biobank*. * p < .05. ** p < .01. *** p < .001. | | | | | | | | | | | |  |  |

| S5 Table.  Adjusted models^a^ of total brain volume (mm^3^) on the interaction of *Toxoplasma gondii* with age, sex, education, and income: Unstandardized coefficients from linear regression | | | |
| --- | --- | --- | --- |
|  | Gray Matter | White Matter | Multivariate *p* |
| Age |  |  |  |
|  |  |  |  |
| *T. gondii* seropositive | 14922 | 66640 |  |
| Age | -3193*** | -1628*** |  |
| Interaction | -337 | -1060 | .203 |
|  |  |  |  |
| ln(p22) | 2929 | 12499 |  |
| Age | -2938*** | -1104 |  |
| Interaction | -112 | -233 | .419 |
|  |  |  |  |
| ln(sag1) | -3216 | 35321 |  |
| Age | -3141* | 600 |  |
| Interaction | -26 | -565 | .089 |
|  |  |  |  |
| Mean of ln(p22) and ln(sag1) | -457 | 31351 |  |
| Age | -3286*** | -1897*** |  |
| Interaction | -91 | -527 | .154 |
|  |  |  |  |
| Sex |  |  |  |
|  |  |  |  |
| *T. gondii* seropositive | -15965* | -10431 |  |
| Female | 23413*** | -9368 |  |
| Interaction | 17298* | 19263* | .022 |
|  |  |  |  |
| ln(p22) | -5864** | -4192 |  |
| Female | 16595 | -18070 |  |
| Interaction | 3322 | 4126 | .255 |
|  |  |  |  |
| ln(sag1) | -5487* | 447 |  |
| Female | 22516 | -1397 |  |
| Interaction | 1279 | -728 | .877 |
|  |  |  |  |
| Mean of ln(p22) and ln(sag1) | -7719** | -2565 |  |
| Female | 28210*** | -4240 |  |
| Interaction | 3033 | 2289 | .663 |
|  |  |  |  |
| Education |  |  |  |
|  |  |  |  |
| *T. gondii* seropositive | -1954 | -3819 |  |
| College degree | -5014 | -2550 |  |
| Interaction | -8037 | 8586 | .232 |
|  |  |  |  |
| ln(p22) | -3190 | -4620* |  |
| College degree | -2887 | -15684 |  |
| Interaction | -1270 | 4607 | .137 |
|  |  |  |  |
| ln(sag1) | -6474* | -2242 |  |
| College degree | -19005 | -16412 |  |
| Interaction | 2535 | 3672 | .503 |
|  |  |  |  |
| Mean of ln(p22) and ln(sag1) | -6489* | -5086 |  |
| College degree | -7569* | -106 |  |
| Interaction | 560 | 5982 | .285 |
|  |  |  |  |
| Income (in 10,000 ₤) |  |  |  |
|  |  |  |  |
| *T. gondii* seropositive | -17817* | -14231 |  |
| Income | -888 | -792 |  |
| Interaction | 2644* | 3336* | .010 |
|  |  |  |  |
| ln(p22) | -7377** | -8669** |  |
| Income | -2525 | -4605* |  |
| Interaction | 713 | 1428** | .007 |
|  |  |  |  |
| ln(sag1) | -5684 | -6187 |  |
| Income | -868 | -6102* |  |
| Interaction | 188 | 1439* | .006 |
|  |  |  |  |
| Mean of ln(p22) and ln(sag1) | -8451* | -10044** |  |
| Income | -58 | 282 |  |
| Interaction | 520 | 1953** | .003 |
| Note: ^a^ Each set of three coefficients represents results from a single model that contains a single interaction. The coefficients reported are the main effect for the *T. gondii* measure, the main effect for the predictor, and the interaction between the *T. gondii* measure and predictor. For example, the first entry in the table is for the interaction between *T. gondii* seropositivity and age with volume of the total brain gray matter as the outcome. Each model adjusts for included age, sex, race, education, income, self-rated health, body-mass index, smoking status, and drinking frequency, although these coefficients are not reported. N = 385. Source: *UK Biobank*. * p < .05. ** p < .01. *** p < .001. | | | |
